# Monoarthrite du genou révélatrice d’une arthropathie tabétique

**DOI:** 10.11604/pamj.2017.26.39.11634

**Published:** 2017-01-30

**Authors:** Zeineb Alaya, Walid Osman

**Affiliations:** 1 Service de Rhumatologie, Hôpital Farhat Hached, Sousse, Tunisie; 2 Service d’Orthopédie, Hôpital Sahloul, Sousse, Tunisie

**Keywords:** Arthrite, genou, tabès, scanner, Arthritis, knee, tabes, CT scan

## Image en médecine

Il s’agit d’un patient âgé de 53 ans, qui a consulté pour une monoarthrite du genou droit évoluant depuis 6 mois sans fièvre, associé à un syndrome inflammatoire biologique. La ponction articulaire a ramené un liquide inflammatoire stérile. Les radiographies du genou de face ( A) et de profil (B) et la TDM du genou (C et D) ont montré une destruction du condyle fémoral interne avec une ostéolyse du rebord médial du plateau tibial interne associé à de multiples constructions osseuses avec présence de fragments intra-articulaires, d’un épanchement intra-articulaire et d’un épaississement de la synoviale. Le diagnostic d'arthropathie tabétique dans sa forme hypertrophique a été retenu devant un antécédent de chancre d’inoculation syphilitique datant de 20 ans, un syndrome radiculocordonal postérieur, les données de l’imagerie et une sérologie syphilitique (TPHA-VDRL) positive dans le sang et le LCR. Le patient a été traité par pénicilline G (24 millions/j) pendant 15 jours. Plus fréquent chez l'homme que chez la femme, le tabès, devenu exceptionnel, se découvre tardivement vers 50 ou 60 ans. L'arthropathie tabétique est une affection neurogène destructrice secondaire à l'infection par le tréponème. Elle concerne 4 à 10% des tabétiques. Le genou est l'articulation la plus souvent atteinte. Les formes de début souvent méconnues miment une gonarthrose banale. L'aspect habituel réalise une forme hypertrophique. Le traitement de l'arthropathie tabétique est essentiellement médical basé sur la pénicilline G.

**Figure 1:**
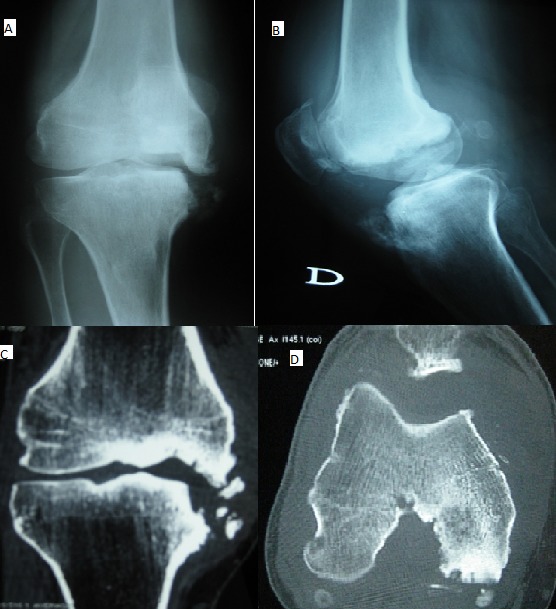
(A,B) radiographie du genou droit de face et de profil: destruction du condyle fémoral interne avec ostéolyse du plateau tibial interne associé à de multiples constructions osseuses; (C,D) scanner du genou droit en coupe sagittale et axiale: destruction du condyle fémoral interne avec ostéolyse du rebord médial du plateau tibial interne associé à de multiples constructions osseuses avec présence de fragments intra-articulaires, d’un épanchement intra-articulaire et d’un épaississement de la synoviale

